# Assessment of hPod measured by fNIRS as an indicator of brain development in preterm infants

**DOI:** 10.1162/IMAG.a.1230

**Published:** 2026-05-18

**Authors:** Anna Shiraki, Hama Watanabe, Takafumi Ushida, Ayano Yanagisawa, Misa Hashimoto, Misae Yamada, Hajime Narita, Takamasa Mitsumatsu, Ryosuke Suzui, Masahiro Kawaguchi, Yuji Ito, Hiroyuki Yamamoto, Tomohiko Nakata, Yoshiaki Sato, Jun Natsume, Gentaro Taga, Hiroyuki Kidokoro

**Affiliations:** Department of Pediatrics, Nagoya University Graduate School of Medicine, Nagoya, Japan; Graduate School of Education, The University of Tokyo, Tokyo, Japan; Department of Obstetrics and Gynecology, Nagoya University Graduate School of Medicine, Nagoya, Japan; Division of Neonatology, Center for Maternal-Neonatal Care, Nagoya University Hospital, Nagoya, Japan; Department of Developmental Disability Medicine, Nagoya University Graduate School of Medicine, Nagoya, Japan

**Keywords:** preterm infants, near-infrared spectroscopy, hPod, sleep states, development

## Abstract

The brain undergoes dramatic development during early life, particularly throughout the third trimester of pregnancy. Preterm infants experience this critical period in the extrauterine environment, which may alter brain development and has recently gained attention for its potential impact on subsequent neurodevelopmental challenges. Brain maturation involves not only neuronal growth but also the development of vascular and neurovascular coupling systems. Hemoglobin phase of oxygenation and deoxygenation (hPod) is a promising biomarker of neurovascular and metabolic functional development. In this study, we investigated differences in hPod values between active sleep (AS) and quiet sleep (QS), as well as regional variation throughout the brain. In all, 240 simultaneous electroencephalography and near-infrared spectroscopy recordings were obtained from 75 preterm infants and 54 term-born infants. The hPod values were calculated as the time-averaged phase difference between oxy- and deoxyhemoglobin signals during AS and QS, respectively. The hPod values exhibited significant positive correlations that were highly consistent within each subject, between AS and QS, and between the bilateral homologous channels. Notably, they exhibited a gradual shift from an in-phase to an anti-phase pattern with an increase in postnatal age prior to term-equivalent age in preterm infants; this pattern emerged earlier in the posterior–temporal regions and was most delayed in the frontal regions. These results suggest that hPod values may have potential as a biomarker of early brain maturation in vulnerable infants, although further validation, particularly in association with neurodevelopmental outcomes including high-risk infants, is required before clinical application.

## 1 Introduction

The perinatal brain undergoes rapid and profound structural and functional changes that form the basis for later cognitive and behavioral development. Monitoring these early processes in the neonatal intensive care unit (NICU) is critical for identifying at-risk infants and informing individualized developmental support during and after NICU care. Electroencephalography (EEG) is widely used as a bedside tool for assessing cortical function (André et al., 2010; Luhmann et al., 2022; Okumura & Kidokoro, 2025; Watanabe et al., 1999; Whitehead et al., 2016). Although a dysmature EEG pattern (i.e., more than 2 weeks immature) raises concerns about future intellectual development, it is difficult to predict outcomes from such a pattern alone (Okumura et al., 2002). To strengthen assessment of brain maturation, additional evaluations, such as vascular and metabolic indices, may be needed to complement EEG.

Near-infrared spectroscopy (NIRS) has gained increasing attention for its ability to monitor cerebral hemodynamics in real time (Hendrikx et al., 2019; Martini et al., 2024; Roche-Labarbe et al., 2007; Siddiqui et al., 2021; Taga et al., 2000). An NIRS-derived metric, the hemoglobin phase of oxygenation and deoxygenation (hPod), has been proposed as a potential indicator of neurovascular development (Watanabe et al., 2017). hPod captures the temporal lag between spontaneous low-frequency oscillations in oxy-hemoglobin (Hb) and deoxy-Hb and may reflect underlying maturational processes of the cerebral vasculature and metabolism. Its simplicity and compatibility with bedside monitoring make it a promising candidate for assessing the brains of neonates in the NICU (Liang et al., 2018; Martini et al., 2024; Taga et al., 2018b; Watanabe et al., 2017).

Watanabe et al. (2017) demonstrated that hPod values transitioned rapidly from an in-phase to an anti-phase pattern in early life, up to 8 weeks of postnatal age (PNA), followed by a more gradual developmental change that persisted during the first 6 months of PNA in preterm and term-born infants. Early preterm infants, born younger than 34 weeks of gestational age (GA), exhibited more anti-phase patterns between 36 and 44 weeks of postmenstrual age (PMA) than late preterm and term-born infants. However, the changes in hPod values toward antiphase patterns at 3–6 months of PNA were slower in early preterm infants than in late preterm and term-born infants, and early preterm infants showed more in-phase patterns. These findings suggest that hPod values may serve as a marker of developmental progression. Rapid changes in hPod values are likely accelerated by factors associated with the environmental transition from the intrauterine to the extrauterine setting, such as maturation of the circulatory system including lungs and heart, and replacement of fetal hemoglobin by adult hemoglobin. In contrast, the gradual decrease in hPod values has been suggested to reflect the maturation of neurovascular coupling (Watanabe et al., 2017).

Certain measures of brain function are known to be sensitive to behavioral state fluctuations. In neonates, there are two major sleep states: active sleep (AS) and quiet sleep (QS). AS and QS are considered to be the precursors of rapid eye movement (REM) and non-REM sleep, respectively, and both sleep states are thought to play important roles in brain development (Knoop et al., 2021; Neukamm et al., 2025; Uchitel et al., 2022). Functional NIRS and EEG studies reported state-dependent differences in newborn functional connectivity (Lee et al., 2020; Shiraki et al., 2024; Tokariev et al., 2019; Uchitel et al., 2023). We earlier showed that behavioral states such as sleep and wakefulness affected sensory cortical processing in term-born infants of PNA greater than 70 days (Taga et al., 2018a). The differences between behavioral states were associated with specific brain regions and/or with certain characteristics of the neural connections. The EEG patterns of AS and QS are very different (André et al., 2010; Dereymaeker et al., 2017; Okumura & Kidokoro, 2025; Tsuchida et al., 2013). Together, the findings suggest that hPod values may differ between AS and QS and/or among cortical regions because developmental hPod changes are likely to be associated with neural activities, at least after a PNA of 2 months (Watanabe et al., 2017). However, the hPod values of the previous study did not distinguish those of AS, QS, or the cortical regions including interhemispheric homologous regions. The key features of hPod patterns during early postnatal brain development and their association with neurodevelopmental outcomes remain poorly understood. As a result, it remains unclear how behavioral states and regional characteristics shape hPod values, whether hPod values differ between interhemispheric homologous regions, and whether these measures predict later neurodevelopmental outcomes. Currently, those values are not readily interpretable, substantially hampering their clinical application to individualized neurodevelopmental monitoring.

In this study, we characterized the developmental trajectory of hPod values in preterm and term-born infants between 31 and 48 weeks of PMA, with a specific focus on sleep-state dependence, regional variation, and interhemispheric symmetry. Furthermore, we sought associations between hPod values and clinical data, such as the GA at birth, sex, and the developmental outcomes. We hypothesized that the hPod patterns would develop from in-phase to anti-phase with increasing PNA in the first life stages of early preterm infants, consistent with previous reports on late preterm and term-born infants (Watanabe et al., 2017). In addition, we hypothesized that the hPod values would differ between AS and QS, potentially reflecting inter-individual variability in the extent of endogenous stimulation during AS. Furthermore, we expected that the hPod values of the temporal and occipital regions would exhibit more pronounced anti-phase patterns than those of other regions, given the proximity of the former regions to the primary sensory cortices. Finally, we proposed that the hPod values after the initial rapid postnatal developmental change might serve as clinically informative markers for predicting future neurodevelopmental outcomes.

## 2 Methods

### 2.1 Participants

We recruited 75 preterm infants admitted to the NICU at Nagoya University Hospital, Japan, between April 2020 and March 2023, as well as 54 healthy term-born infants born at the hospital between October 2021 and March 2023. The study was approved by the Ethics Committee of Nagoya University Hospital (approval no. 2019-0506 and 2021-0298). Written informed consent was obtained from the parents of all infants.

The inclusion criteria for preterm infants were as follows: GA at birth <35.0 weeks, absence of major congenital malformations, and not being intubated at the time of recording (infants receiving respiratory support such as nasal continuous positive airway pressure, high-flow nasal cannula, or nasal oxygen therapy were included). Neonates with severe brain injuries, such as cystic periventricular leukomalacia or grade III or IV intraventricular hemorrhage, were excluded. Preterm infants were categorized into early and late based on the GA at birth (<34 weeks and 34–35 weeks respectively).

The inclusion criteria for term-born infants were as follows: GA at birth of 37.0–41.0 weeks, birth weight above the 10^th^ percentile for GA at birth, 5 min Apgar score ≥8, no major congenital malformations, absence of clinical symptoms at the time of recording, no significant concerns at the 1-month check-up (passed metabolic and hearing screening tests), and maternal history free from hypertensive disorders of pregnancy, gestational diabetes mellitus requiring insulin therapy, and severe psychiatric illness.

Additional two preterm infants and five term-born infants who had met the inclusion criteria were excluded due to insufficient NIRS data.

### 2.2 Procedure

For preterm infants, EEG–functional NIRS (fNIRS) recordings were conducted at intervals of at least 10 days, continuing until discharge to home or transfer to another hospital. There was no maximum limit on the number of recordings. For term-born infants, they were conducted once between days 1 and 9 after birth.

Recordings were performed after the infants fell asleep naturally and continued until both AS and QS periods were captured. This typically required more than 30 min, consistent with the ultradian sleep cycle observed in neonates, which is approximately 50–60 min (range, 30–70 min) (Dereymaeker et al., 2017). EEG and NIRS data were obtained simultaneously using different instruments, and synchronization was achieved by manually inputting transistor-transistor logic pulses via a button switch to both devices for post-processing alignment.

Polygraphy recordings were obtained using EEG (EEG-1200; Nihon Kohden, Tokyo, Japan), electrooculography, a monitor of abdominal respiratory movement, a chin electromyogram, an electrocardiogram, and video-recording. A minimum of eight EEG electrodes (Fp1, Fp2, C3, C4, O1, O2, T3, and T4) were placed according to the international 10–20 system, with a 0.002-s time resolution.

**Fig. 1.**
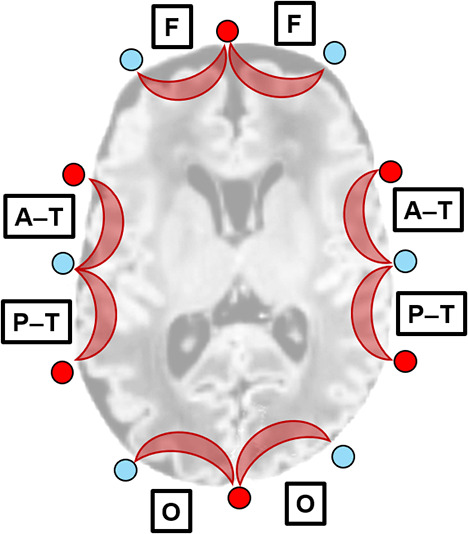
Placement of NIRS channels. Red and blue circles: Sources and detectors, respectively. Squares: NIRS channels and their corresponding regions. F, frontal; A–T; anterior–temporal; P–T, posterior–temporal; and O, occipital. Each region included two bilateral homologous channels.

An eight-channel NIRS device (ETG-100; Hitachi Medical Corporation, Tokyo, Japan) was placed around the infant’s head using a headband, covering the frontal, bilateral temporal, and occipital regions (Fig. 1). Six sources and six detectors were arranged in a single row on the headband, just above the Fp1, T3, O1, O2, T4, and Fp2 EEG electrodes. Each pair of adjacent sources and detectors formed one measurement channel, totaling eight channels. The distance between each source and detector was 2 cm. The NIRS instrument generated two wavelengths of near-infrared light (780 and 830 nm) and measured the time courses of relative changes in oxy- and deoxy-Hb concentrations with a time resolution of 0.1 s. The methodology was the same as that of our previous publication (Shiraki et al., 2024).

### 2.3 Classification of vigilance states

Vigilance states were manually classified by two board-certified child neurologists (A.S. and H.K.) using EEG polygraphy data, according to the criteria of the American Clinical Neurophysiology Society and previous reports describing the characteristics of vigilance states in infants (André et al., 2010; Dereymaeker et al., 2017; Grigg-Damberger, 2016; Tsuchida et al., 2013). The vigilance states were classified into six categories: AS, QS, indeterminate sleep [IS], transitional sleep, awake, and unclassifiable because of artifacts or data defects. The states were scored every 30 s using the EEG patterns, the REMs, respiratory patterns, and eye-opening/closing status. Although a chin electromyogram was a component of EEG polygraphy, this was not used when classifying vigilance states because it readily dropped away when infants moved. AS and QS were scored only when all four indices were consistent with the characteristic features of each state. During AS, the EEG exhibits continuous or semi-continuous activity in the preterm period and low-voltage irregular or mixed patterns at term-equivalent age. AS also features more frequent REMs than QS, an irregular respiratory pattern, and closed eyes. In contrast, during QS, the EEG exhibits *tracé discontinu* patterns in the preterm period and high-voltage slow or *tracé alternant* patterns at term-equivalent age, with fewer REMs, regular respiration, and closed eyes (Supplementary Fig. S1). IS was scored when not all of the four indices fulfilled the AS, QS, or awake criteria, or when the AS or QS durations were only 30 s. Transitional sleep was associated with an IS intermediate between the different vigilance states. ‘Awake’ was scored when the infant kept his/her eyes open during the 30-s window. When the classifications differed between the two scorers, the final decision was determined via discussion. Based on the final results for all four indices, each vigilance state was classified in line with the above definitions. Given that measurement over at least several minutes is required to assess slow oscillation (0.05–0.1-Hz), and as IS and awake data were limited (Table 1), only data segments classified as AS or QS ≥2 min in duration within recordings in which the total duration of such segments was ≥3 min were included in further analysis.

**Table 1.** Durations of segments of each vigilance state in early preterm, late preterm, and term-born infants, and hPod data acquired during both active and quiet sleep.

	Preterm infants (n = 167)		Term-born infants (n = 54)
	Early (n = 150)	Late (n = 17)	
Total recording duration (min)	66.75 (42.0–99.5)	77.5 (60.0–103.0)	73.75 (47.5–96.0)
Active sleep			
Total duration (min)	31.25 (6.0–67.0)	32.0 (17.5–50.0)	36.0 (15.0–67.5)
Analytical duration (min)	24.0 (3.0–62.5)	24.5 (15.0–42.0)	34.0 (14.0–67.5)
Duration excluded because of PB (min)	2.0 (0.0–40.0)	0.0 (0.0–12.5)	0.0 (0.0–13.5)
Quiet sleep			
Total duration (min)	20.25 (5.5–38.0)	26.0 (10.0–50.5)	24.0 (12.0–49.0)
Analytical duration (min)	17.0 (3.0–37.0)	24.5 (7.0–49.5)	23.25 (10.5–48.0)
Duration excluded because of PB (min)	0.0 (0.0–21.0)	0.0 (0.0–3.5)	0.0 (0.0–2.0)
Indeterminate sleep (min)	4.0 (0.0–23.5)	5.0 (0.0–18.5)	3.5 (0.0–13.0)
Transitional sleep (min)	3.5 (0.0–22.0)	5.0 (1.0–11.5)	3.5 (0.5–12.5)
Awake (min)	0.0 (0.0–23.5)	3.0 (0.0–30.5)	0.0 (0.0–10.0)
Excluded because of artifacts (min)	3.25 (0.0–29.0)	7.0 (0.0–38.5)	2.0 (0.0–12.0)

Values are medians (ranges).

The total duration is the sum of all segmental durations, including those less than 2 min, irrespective of the presence or absence of periodic breathing (PB).

The analytical duration and that excluded because of PB are the cumulative durations of all segments without and with PB, respectively, ≥2 min in duration in each sleep state.

PB, periodic breathing.

### 2.4 Definition and detection of periodic breathing

Periodic breathing (PB), characterized by repetitive short respiratory cycles alternating with pauses (Berry et al., 2012), is commonly observed in infants. We previously showed (Shiraki et al., 2024) that oxy- and deoxy-Hb signals across the eight NIRS channels vary by the duration of PB apnea (Supplementary Fig. S2). Therefore, in this study, we analyzed only the hemodynamic data from segments lacking PB. The 30-s segments that followed those containing PB were also excluded because the effect of PB on hemodynamic signals can persist throughout the immediately following segment. We used an automated algorithm to detect PB (Shiraki et al., 2024) and also reviewed the data manually to confirm that PB detected by the algorithm met the standard definition: three or more episodes of central apnea lasting >3 s separated by ≤ 20 s of normal breathing (Berry et al., 2012). The automated algorithm uses the envelope data of abdominal respiratory movement filtered from 0.3–2.0 Hz, and identifies PB apnea when the mean amplitude within a sliding window of 3 s is <0.35 fold the mean amplitude within the nearest 6-min reference window. The algorithm classified segments as including PB if they contained ≥3 episodes of repeated apneic intervals 3–20 s in duration, separated by breathing intervals of 3–20 s (Shiraki et al., 2024).

### 2.5 Preprocessing of NIRS data

The preprocessing procedures, including the exclusion of data affected by motion artifacts, PB, or technical issues and subsequent baseline correction, have been described (Shiraki et al., 2024). A motion artifact was defined when the difference in the summed oxy- and deoxy-Hb signals was >0.15 mM·mm between the mean values of four successive samples and the next four samples. A measurement error was identified when no signal change was noted over eight consecutive samples in each channel. If five or more channels fulfilled these criteria, all data were excluded at those time points during subsequent analyses. In addition, all data of a given channel were excluded if the signals were excessively noisy (mean difference in the absolute value of the summed oxy- and deoxy-Hb signals between two consecutive samples >0.2 mM·mm), or only <30% of all NIRS data were available for analysis. In terms of PB, excluded segments were chosen as described above. NIRS signals are relative changes calculated as differences from a baseline defined by the initial light intensity. However, when motion artifacts are excluded, the baseline can shift, and this may be misinterpreted as a true signal change. To address this, the baseline signal after exclusion was corrected using the mean values of 10 samples from segments immediately before and after the excluded portion. We analyzed recordings that included hemodynamic data from 7 or 8 NIRS channels.

After identifying segments free from artifacts and periodic breathing, and correcting for baseline signals, the oxy- and deoxy-Hb signals were band-pass filtered within the 0.05–0.1-Hz frequency range. This range is associated with neurovascular coupling; thus, we did this to remove physiological (e.g., heartbeat, respiration) and measurement (e.g., slow signal drift) noise.

### 2.6 Calculation of hPod

hPod values were calculated using the method of Watanabe et al. (2017). To extract the instantaneous phases of the signals [*x(t)*], a Hilbert transformation was applied to the preprocessed hemodynamic data [*y(t)*]. The instantaneous phase θ*(t)* for the oxy- and deoxy-Hb signals was calculated as follows:

$$\theta \left(t\right)=arctan\left(\frac{y\left(t\right)}{x\left(t\right)}\right).$$

Then, the phase difference between the instantaneous phases of the oxy- and deoxy-Hb signals [ϕ*(t)*] was computed for each channel as:

$$\;\phi (t)=\theta_{oxy}(t)-\theta_{deoxy}(t).$$

The hPod was defined as the time-averaged phase difference between the oxy- and deoxy-Hb signals for each record. The calculation is shown below, where *r* is the phase difference:

$$\;re^{ihPod}=\;\frac{1}{T}\int_{0}^{T}e^{i\phi \left(t\right)}dt.$$

An all-channel-averaged value of hPod was calculated as the vectorial sum of the hPod values from all available channels (seven or eight). An angular value >1.5π indicates a pattern close to in-phase (corresponding to 0 or 2π) whereas a value <1.5π indicates a pattern close to anti-phase (corresponding to π).

hPods were calculated separately for AS and QS analytical segments, as mentioned above.

### 2.7 Neurodevelopmental assessment of preterm infants

Psychologists blinded to the NIRS data results evaluated the developmental quotients (DQs) of preterm infants at 18 months of corrected age using the Kyoto Scale of Psychological Development (KSPD) 2001 or 2020 (Ikuzawa et al., 2002; Working Group of the Kyoto Scale of Psychological Development, 2020). This is a major tool used in the follow-up of preterm infants in Japan, and KSPD scores correlate strongly with those on the Bayley Scales of Infant and Toddler Development, third edition (Bayley-III) (Kono et al., 2016).

### 2.8 Statistical analysis

First, we investigated whether the hPod values were influenced by the vigilance states or laterality. If not, we used a subset of the hPod values in subsequent evaluations, for simplicity.

Circular statistics were used to assess the correlations between AS and QS hPod values and those of bilateral homologous channels. As hPod values are angular data (i.e., 0 = 2π), the circular–circular correlation coefficient ρ was used for these evaluations.

As shown previously (Watanabe et al., 2017), hPod values would be expected to be influenced by the PNA rather than the PMA at the time of recording. To confirm that this was the case for our current dataset that included recordings at younger PMAs, we assessed the hPod values in the contexts of both PMA and PNA. The all-channel-averaged data from early preterm infants were subjected to curve regression because early preterm infants had broader PMA and PNA at the time of recording than late preterm and term-born infants. As all data were distributed between π and 2π, they were regarded as linear values.

Next, we assessed differences in hPod values among groups based on PMA and PNA at the time of recording. For PMA at the time of recording, we distinguished four groups: preterm infants recorded at 33–34 weeks, 35–36 weeks, and 37–41 weeks PMA, and term-born infants. To assess differences based on PNA, we categorized recordings from early preterm infants into three groups: PNA < 40 days, 40–79 days, and ≥80 days. Data from late preterm infants and term-born infants were not used when comparing differences based on PNA, because almost all such infants were recorded at ages <40 days of PNA. When multiple data points were available for the same infant within a categorized group, the recording obtained at the younger age was selected, because it was reasonable to compare hPod values at similar recording ages given the overall data distributions in terms of PMA and PNA at the time of recording. The mean hPod value of each group was calculated via vector summation and compared using circular statistics. As hPod values are angular data, the group-level statistical analyses employed circular statistical methods that differ from those typically used for scalar data. For both analyses, the Watson–Williams test, corresponding to one-factor ANOVA, was performed to compare the mean hPod angles among the groups. Subsequently, *post-hoc* tests were conducted using the Tukey–Welsch procedure.

To evaluate developmental changes across different regions, we compared the mean hPod angles among the measured regions, (frontal, anterior–temporal, posterior–temporal, and occipital) (Fig. 1) for each of the three PNA groups. The hPod values averaged from bilateral channels of the region were used. When the hPod value was available from only one side, that value was used. Mixed ANOVA (3 [PNA age] × 4 [region]) was performed. Because the region values were obtained from the same subjects, the Watson–Williams test is not suitable for repeated-measures data. Therefore, the cosine of the hPod values was calculated and treated approximately as scalar data. Then, a standard 3 (age: between-subjects) × 4 (region: within-subjects) mixed-design ANOVA was conducted. *Post-hoc* tests were performed for comparisons among the age groups after Bonferroni corrections and those among the regional groups that employed the FDR method.

Finally, we compared the hPod values between clinical groups according to GA at birth (<28 weeks vs. ≥28 weeks), sex, and DQ at 18 months corrected age (<85 vs. ≥85) using data recorded after a PNA of 40 days. The Watson–Williams tests were performed separately for each comparison.

## 3 Results

### 3.1 Clinical characteristics of infants and EEG–fNIRS recordings

In total, 186 recordings from 75 preterm infants and 54 recordings from 54 term-born infants, for whom hPod data were available during either AS or QS were included. There were 29 females (39%) among the preterm and 30 (56%) among the term-born infants. The median (range) GA at birth, the PMA, and the PNA at the time of recording were 32.0 (24.6–34.9) weeks, 36.6 (31.0–48.1) weeks, and 32.5 (2–148) days, respectively, for preterm infants; and 38.7 (37.0–40.7) weeks, 39.2 (37.6–41.3) weeks, and 2 (1–9) days for term-born infants. The median (range) number of repeated recordings per infant was 2 (1–7) in preterm infants (Supplementary Fig. S3). More detailed information on the recording is given in Table 2.

**Table 2.** Characteristics of the recordings.

	Preterm infants (n = 186)		Term-born infants (n = 54)
	Early (n = 168)	Late (n = 18)	
GA at birth (weeks)	30.9 (24.6–33.9)	34.4 (34.0–34.9)	38.7 (37.0–40.7)
PMA at the time of recording (weeks)	36.6 (31.0–48.1)	36.4 (34.3–43.3)	39.2 (37.6–41.3)
PNA at the time of recording (days)	35.5 (3–148)	16.5 (2–64)	2 (1–9)
AS (8-ch/7-ch/no data^#^)	158 / 5 / 5	16 / 2 / 0	51 / 3 / 0
QS (8-ch/7-ch/no data^#^)	151 / 4 / 13	15 / 2 / 1	51 / 3 / 0
AS and QS (8-ch/7-ch/no data^#^)	146 / 4 / 18	15 / 2 / 1	51 / 3 / 0

Values are shown as medians (ranges) or numbers.

All recordings included hPod values from either or both AS and QS periods.

The bottom three rows indicate the number of recordings for which hPod values are available, along with the number of available channels.

# No data indicates either that data were available from 6 channels or fewer, or that the duration of AS or QS segments was insufficient to meet the inclusion criteria (≥2 min in duration within recordings in which the total duration of such segments was ≥3 min).

AS, active sleep; GA, gestational age; PMA, postmenstrual age; PNA, postnatal age; and QS, quiet sleep.

### 3.2 hPod values in AS and QS

**Fig. 2.**
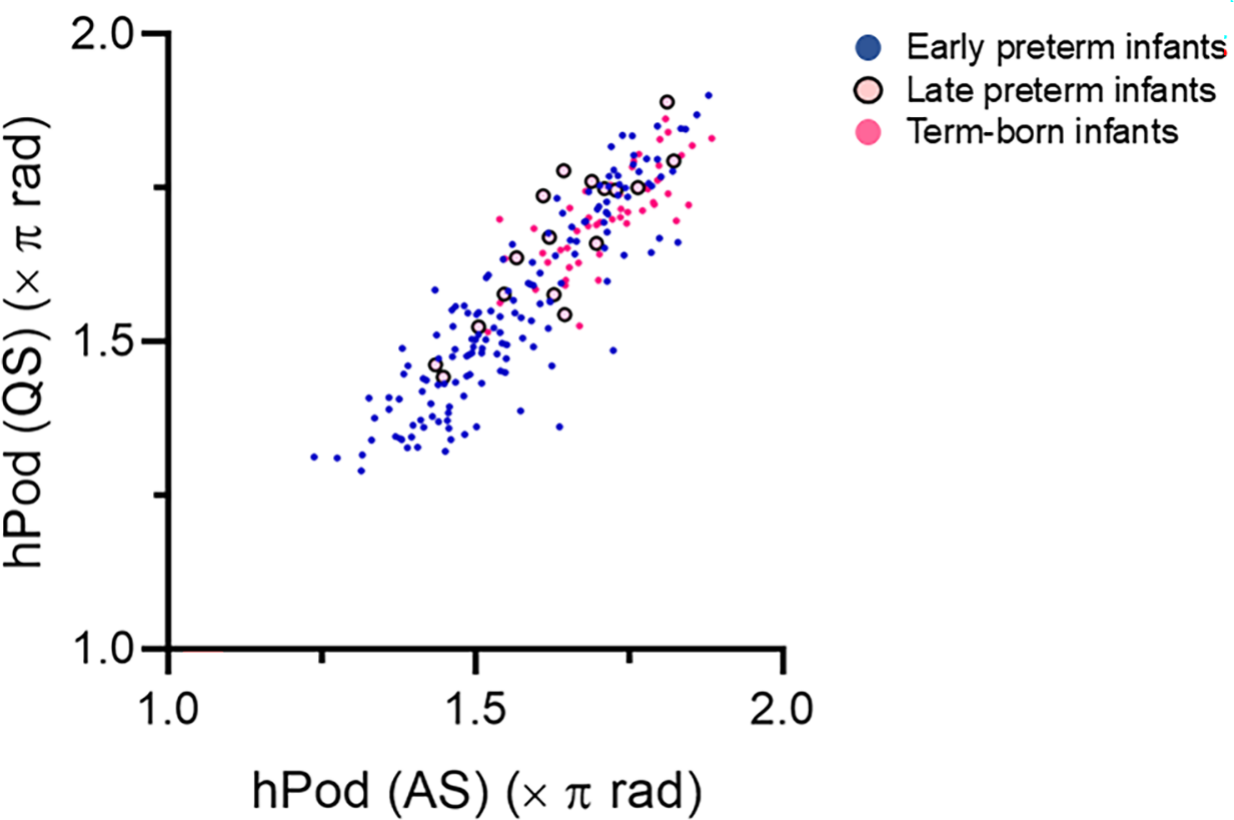
Scatter plot of all-channel-averaged hPod values during active sleep (AS) and quiet sleep (QS), based on a total of 221 recordings from early preterm, late preterm, and term-born infants. Each recording included both AS and QS. The correlation was assessed using the circular–circular correlation coefficient.

We compared the hPod values between AS and QS using 167 recordings from preterm infants and 54 from term-born infants that contained both AS and QS segments within a single recording. All-channel-averaged hPod values showed a significant positive correlation between AS and QS (Fig. 2, ρ = 0.91, *t* (219) = 31.48, *p* < 0.001). This showed that, although the hPod values varied across individuals, they remained highly consistent between AS and QS within each subject. Positive correlations and close agreements among the hPod values between AS and QS were apparent across all four regions (Supplementary Fig. S4, ρ = 0.87, *t* (219) = 25.73, *p* < 0.001 for frontal; ρ = 0.79, *t* (219) = 18.90, *p* < 0.001 for anterior–temporal; ρ = 0.86, *t* (219) = 24.41, *p* < 0.001 for posterior–temporal; and ρ = 0.79, *t* (219) = 18.89, *p* < 0.001 for occipital). Therefore, only the hPod values during QS were used for subsequent analyses, considering that QS involves less body movement compared to AS and is less likely to affect hemodynamic data.

### 3.3 Laterality differences in hPod values

**Fig. 3.**
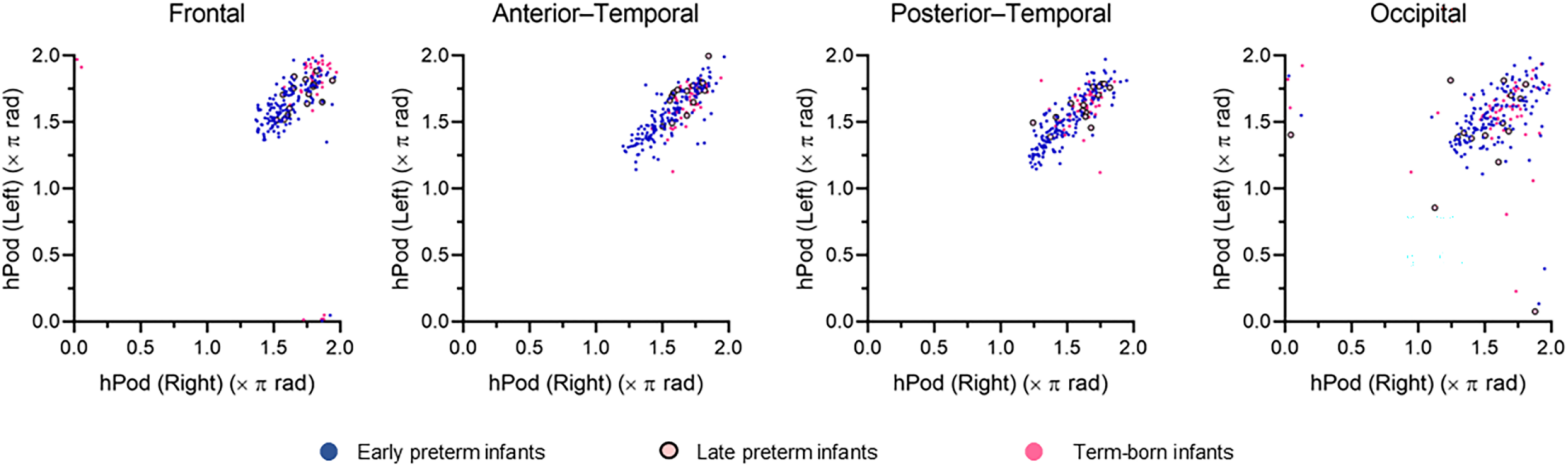
Scatter plots of hPod values during quiet sleep from bilateral homologous channels in the frontal, anterior–temporal, posterior–temporal, and occipital regions, based on a total of 217 recordings from early preterm, late preterm, and term-born infants with eight-channel data. Note that hPod values are circular, with 0π and 2π representing the same value. The correlation was assessed using the circular–circular correlation coefficient.

To assess potential laterality differences in hPod values, we compared hPod values during QS between the bilateral homologous regions in each of the bilateral frontal, anterior–temporal, posterior–temporal, and occipital channels. We used 166 recordings from preterm infants and 51 from term-born infants, all of which included full-channel hPod data, because comparisons would not be valid if any channel lacked an hPod value. In all regions, hPod values from bilateral homologous channels demonstrated strong positive correlations (Fig. 3, ρ = 0.79, *t* (215) = 18.67, *p* < 0.001 for frontal; ρ = 0.81, *t* (215) = 20.34, *p* < 0.001 for anterior–temporal; ρ = 0.83, *t* (215) = 21.43, *p* < 0.001 for posterior–temporal; and ρ = 0.77, *t* (215) = 17.49, *p* < 0.001 for occipital) and were remarkably similar between the left and right channels within each subject. Based on these findings, the averaged hPod values of bilateral homologous channels were used in subsequent analyses to evaluate regional values.

### 3.4 Patterns of hPod values

**Fig. 4.**
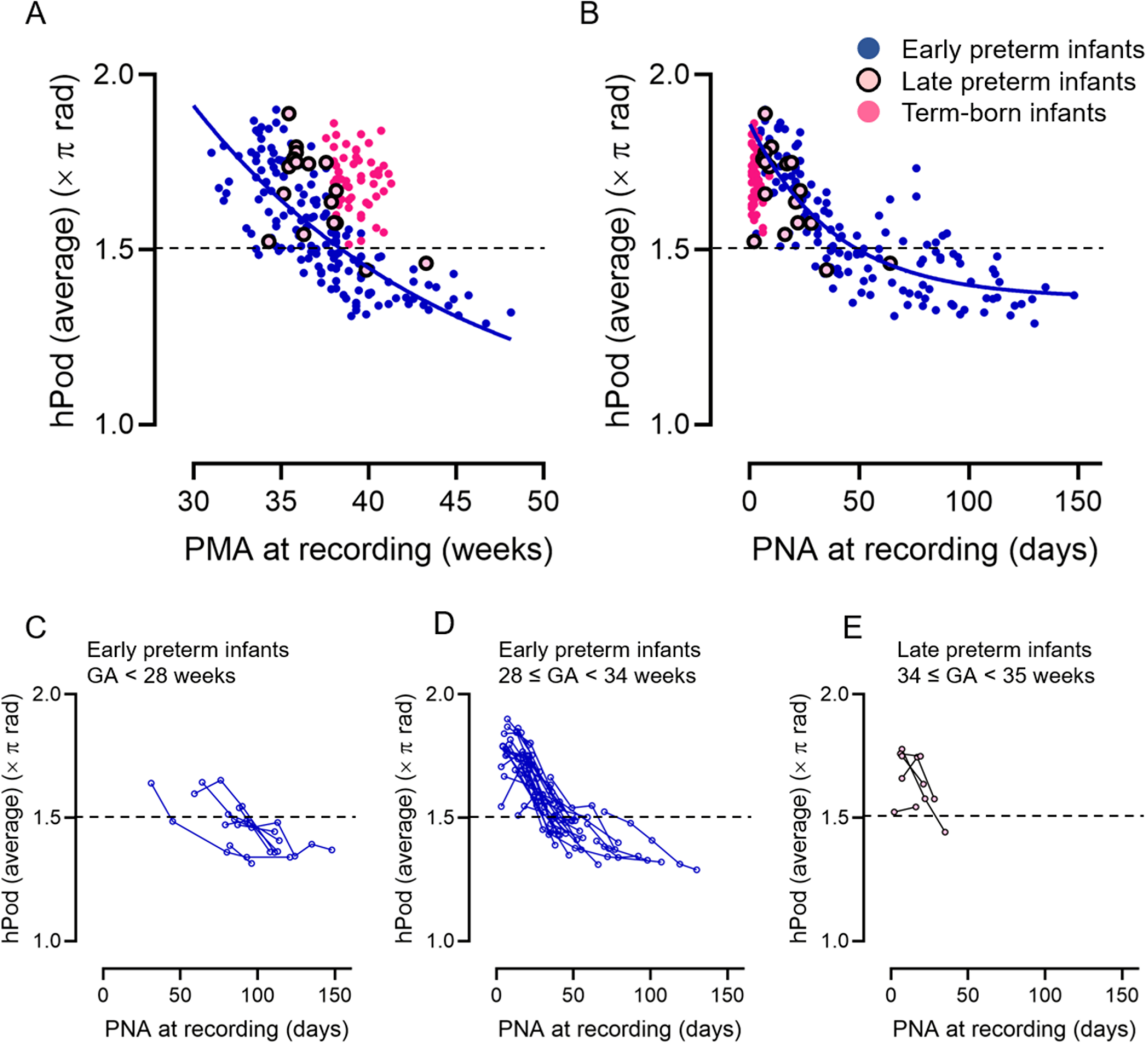
(A, B) Changes in all-channel-averaged hPod values as a function of postmenstrual age (PMA) (weeks, A) and postnatal age (PNA) (days, B) at the time of recording. Blue dots represent early preterm infants (n = 155; gestational age [GA] at birth <34 weeks), pink circles late preterm infants (n = 17; GA at birth 34–35 weeks), and magenta dots term-born infants (n = 54). Blue solid lines indicate the logarithmic trends in early preterm infants, assuming that hPod values are linear variables (R^2^ = 0.60, *p* < 0.001 for PMA; R^2^ = 0.65, *p* < 0.001 for PNA). (C–E) Longitudinal changes of all-channel-averaged hPod values as a function of PNA at the time of recording (days). Infants for whom records were available at two or more time points were included. (C) Twenty-eight recordings from eight early preterm infants with GAs at birth <28 weeks, (D) A total of 111 recordings from 39 early preterm infants of birth GAs 28–33 weeks, and (E) 12 recordings from five late preterm infants whose GAs at birth were 34–35 weeks. The circle colors are those of the groups in A and B. Black dotted lines indicate 1.5π, which is the midpoint between the in-phase (0 or 2π) and anti-phase (π) patterns.

**Fig. 5.**
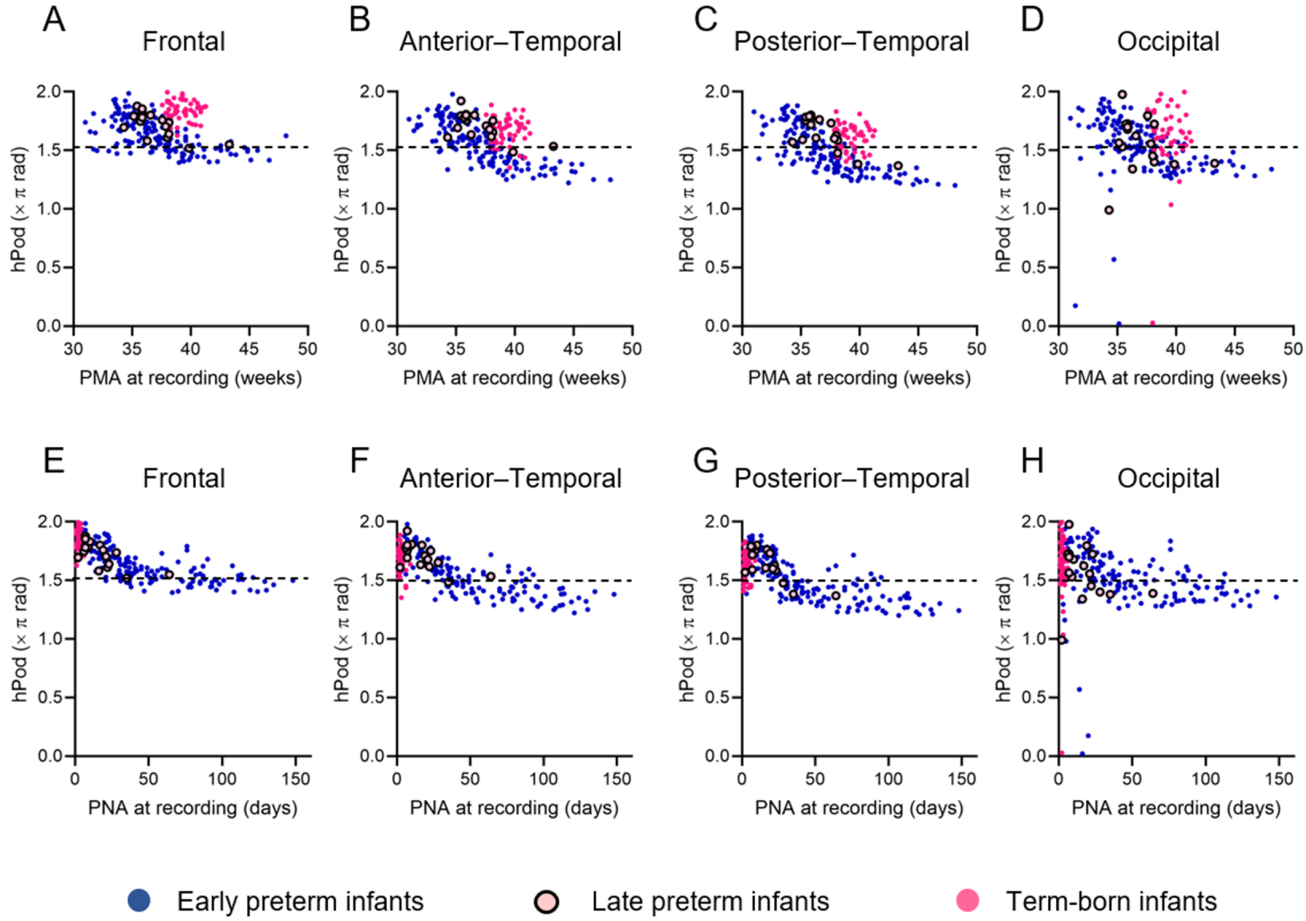
Changes in hPod values as a function of postmenstrual age (PMA) and postnatal age (PNA) at the time of recording in different brain regions. (A–D) show data plotted against PMA (weeks) and (E–H) data plotted against PNA (days). The hPod values are depicted as follows: frontal channels (A, E), anterior–temporal channels (B, F), posterior–temporal channels (C, G), and occipital channels (D, H). Blue dots represent early preterm infants (n = 155; gestational age at birth <34 weeks), pink circles late preterm infants (n = 17; gestational age at birth 34–35 weeks), and magenta dots term-born infants (n = 54). Black dotted lines in all figures indicate 1.5π, which is the midpoint between the in-phase (0 or 2π) and anti-phase (π) patterns.

Figure 4 presents scatter plots of all-channel-averaged hPod values during QS as a function of age at the time of recording: 155 recordings from early preterm infants, 17 from late preterm infants, and 54 from term-born infants. Figure 4A and B show that the hPod values shifted from in-phase (>1.5π) to anti-phase (<1.5π) as a function of PMA and PNA respectively. As shown in Figure 4A, the hPod values of late preterm and term-born infants were higher than those of early preterm infants at the same PMA. In contrast, Figure 4B shows that, although term-born infants exhibited marked variability in terms of the hPod values immediately after birth, both early and late preterm infants demonstrated a rapid shift from in-phase to anti-phase values by around 40 days of PNA. The longitudinal dataset also exhibited a rapid shift from in-phase to anti-phase patterns in the first 40 days of PNA (Fig. 4C, D and E). The same trend was observed in each brain region (Fig. 5). Overall, the results indicate that age-related changes in hPod differ by the GA at birth when aligned using PMA but, when aligned employing PNA, preterm infants—regardless of the GA at birth—exhibit a rapid shift from in-phase to anti-phase within the first 40 days after birth.

### 3.5 Differences in hPod values across PMA groups of preterm and term-born infants

**Fig. 6.**
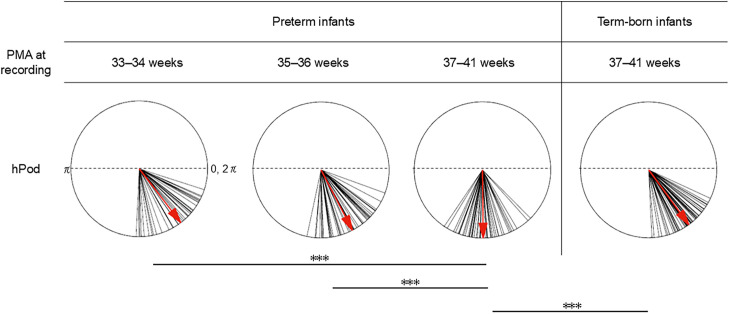
Comparison of all-channel-averaged hPod values among groups based on postmenstrual age (PMA) at the time of recording. Preterm infants were categorized into three groups: 33–34 weeks (n = 36), 35–36 weeks (n = 43), and 37–41 weeks (n = 47). Term-born infants recorded between 37–41 weeks were treated as an independent group (n = 54). If multiple recordings from the same infant fell within a group, only the youngest recording was included. The right edge of dotted lines shows in-phase (0, 2π) values, while the left shows anti-phase (π) values. Solid lines represent the hPod values of each recording during quiet sleep, and red arrows indicate group-averaged values, computed as the vector summation of all-channel-averaged hPod values within each group. Group comparisons were conducted using the Watson–Williams test, followed by the Tukey–Welsch *post-hoc* procedure. ***p < 0.001.

To assess how hPod values change with PMA at the time of recording, hPod values across three preterm recording groups based on the PMA at the time of recording and one term-born group were compared. The all-channel-averaged hPod values exhibited a gradual shift from in-phase to anti-phase patterns across the preterm groups at 33–34, 35–36, and 37–41 weeks PMA at the time of recording, consistent with the scatter plots shown in Figure 4. Statistical analyses revealed a significant difference in mean all-channel-averaged hPod values among the various groups (Fig. 6, *F*[3, 179] = 38.50, *p* < 0.001). The mean hPod value in the 37–41 weeks PMA group was less than those in other three groups including term-born group (*p* < 0.001). This indicates that changes in hPod values do not follow a simple trend of decreasing with increasing PMA at the time of recording. Notably, although the PMA at the time of recording was greater in term-born infants than in term-equivalent preterm infants (37–41 weeks PMA) (Supplementary Table S1), the hPod values were significantly higher in term-born infants. Similar trends were observed across all four regions (Supplementary Fig. S5). Various significant characteristics such as the GA at birth, the proportion of female infants, and the QS analytical duration did not differ among the groups, except that the term-born infant group exhibited a significant older GA at birth and a longer analytical duration during QS than the other three preterm infant groups (Supplementary Table S1).

### 3.6 Comparison of hPod values across PNA groups of preterm infants

**Fig. 7.**
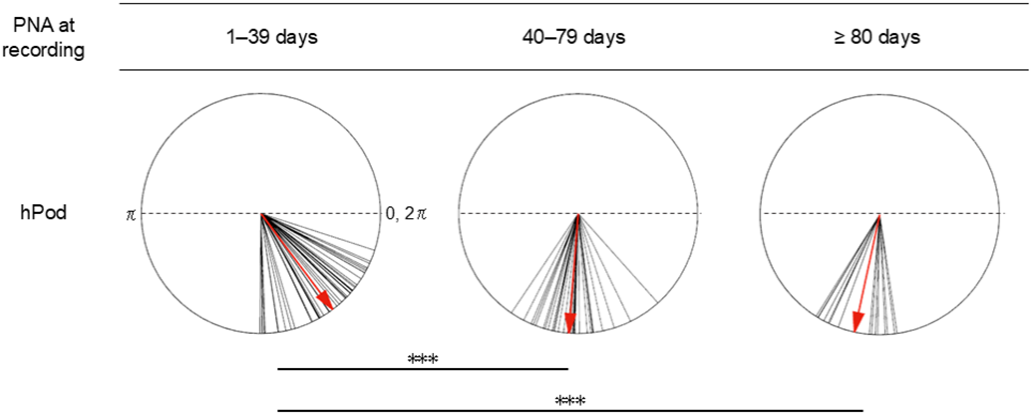
Comparison of all-channel-averaged hPod values among groups based on postnatal age (PNA) at the time of recording in early preterm infants. Recordings were categorized into three groups: 1–39 days (n = 46), 40–79 days (n = 30), and ≥80 days (n = 16). If multiple recordings from the same infant fell within a group, only the youngest recording was included. The right edge of dotted lines shows in-phase (0, 2π) values, while the left shows anti-phase (π) values. Solid lines represent the hPod values of each recording during quiet sleep, and red arrows indicate group-averaged hPod values, computed as the vector summation of all-channel-averaged hPod values within each group. Group comparisons were conducted using the Watson–Williams test, followed by the Tukey–Welsch *post-hoc* procedure. ***p < 0.001.

To investigate how hPod values changed by the PNA, the all-channel-averaged hPod values from early preterm recordings were compared among three groups categorized by the PNA at the time of recording in 40-day intervals, because hPod values rapidly decreased during the first 40 days of PNA in early preterm infants of this cohort (Figs. 4 and 5). There were significant differences in the mean all-channel-averaged hPod values among the three infant groups (Fig. 7, *F*[2, 91] = 65.98, *p* < 0.001). Consistent with the scatter plots (Fig. 4), the 1–39 days group showed a significant in-phase pattern compared to the 40–79 and ≥80 days groups (*p* < 0.001). Although the proportion of female infants and the analytical duration during QS did not differ among the groups, the GA at birth was significantly lower in groups of higher PNA (Supplementary Table S2).

### 3.7 Comparison of hPod values among measurement regions

**Fig. 8.**
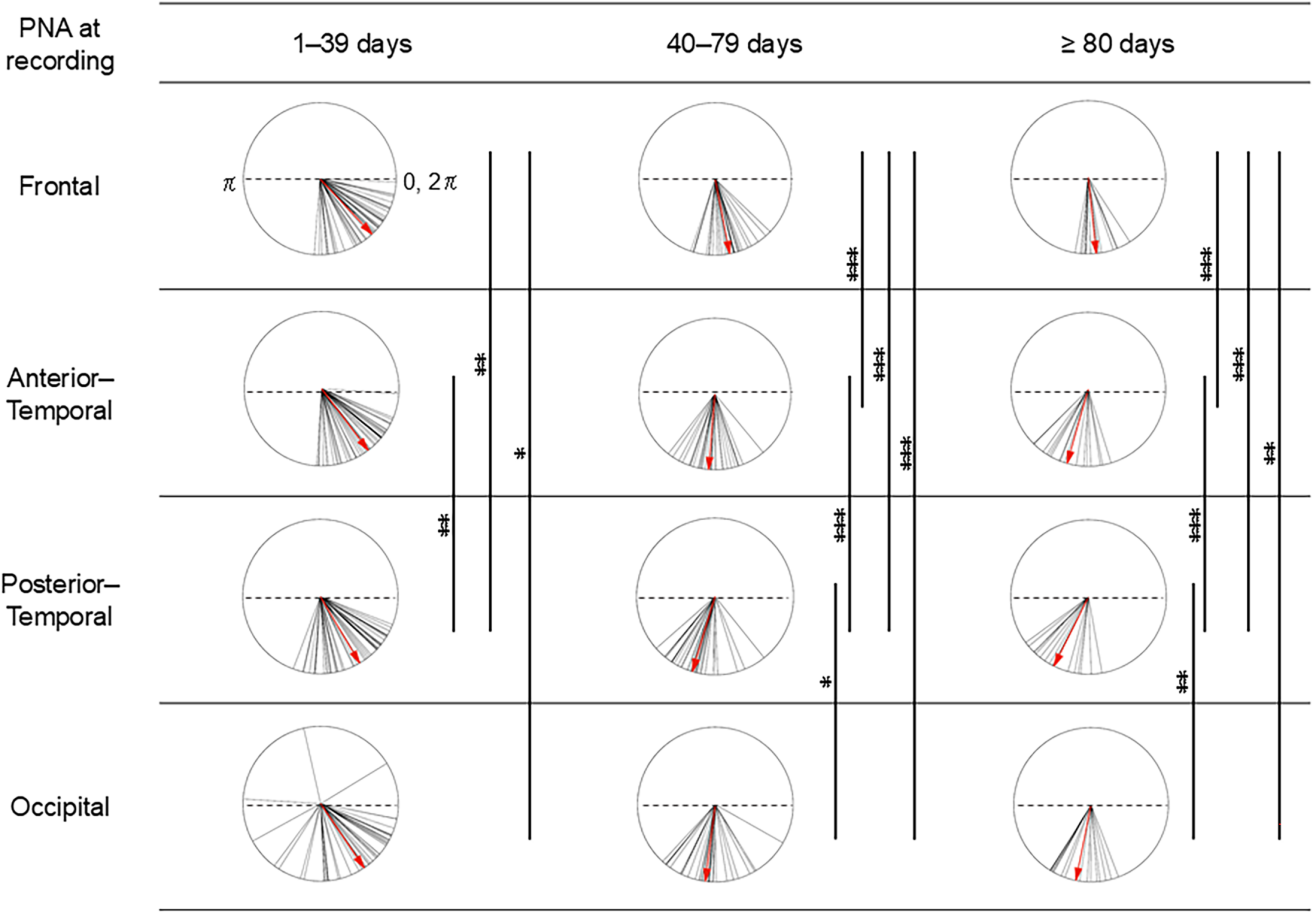
Comparison of regional differences in hPod values among groups based on postnatal age (PNA) at the time of recording in early preterm infants. Group categorization is the same as in Figure 7. The right edge of dotted lines shows in-phase (0, 2π) values, while the left shows anti-phase (π) values. Solid lines represent hPod values averaged across bilateral homologous channels in each recording during quiet sleep in the frontal, anterior–temporal, posterior–temporal, and occipital regions. Red arrows indicate group-averaged hPod values, computed as the vector summation of the bilaterally averaged hPod values within each group. Group comparisons were conducted using mixed ANOVA. *p < 0.05, **p < 0.01, ***p < 0.001. The hPod values in the 1–39-day PNA group differed from those of the 40–79-day PNA group and the ≥80-day PNA group in all regions (all *p* < 0.001).

Regional hPod values were also assessed in the PNA groups of early preterm infants. For this, the cosines of the hPod values were calculated. A mixed ANOVA revealed a significant main effect of PNA group (Fig. 8, *F*[2, 89] = 63.97, *p* < 0.001), a significant main effect of region (*F*[3, 267] = 27.07, *p* < 0.001), and a significant interaction between PNA group and region (*F*[6, 267] = 3.56, *p* < 0.01). In terms of the interaction, a simple main effect of the PNA group was significant in each region (*F*[2, 89] = 33.75 for the frontal, *F*[2, 89] = 62.65 for the anterior–temporal, *F*[2, 89] = 60.13 for the posterior–temporal, and *F*[2, 89] = 19.01 for the occipital, all *p* < 0.001). *Post-hoc* comparisons revealed that the hPod values in the 40–79-day PNA group and the ≥80-day PNA group shifted to anti-phase patterns compared to those observed in the 1–39-day PNA group in all regions (all *p* < 0.001). No significant difference in the hPod value between the 40–79-day PNA group and the ≥80-day PNA group was observed in any region.

A significant simple main effect of region was also significant in each PNA group (*F*[3, 135] = 4.47, *p* < 0.01 for the 1–39-day PNA group, *F*[3, 87] = 24.16, *p* < 0.001 for the 40–79-day PNA group, and *F*[3, 45] = 18.65, *p* < 0.001 for the ≥80-day PNA group). *Post-hoc* comparisons in the 1–39-day PNA group showed that the hPod values in the frontal and anterior–temporal regions were significantly greater (with more in-phase patterns) than those in the posterior–temporal region (all *p* < 0.01), and that the hPod of the frontal region was significantly higher (more in-phase patterned) than that of the occipital region (*p* < 0.05). In the 40–79-day PNA group and the ≥80-day PNA group, *post-hoc* comparisons revealed that the hPod values in the frontal region were significantly greater (more in-phase patterned) than those in other regions (*p* < 0.001 for all regions in the 40–79-day PNA group, *p* < 0.001 for the anterior–temporal and the posterior–temporal, and *p* < 0.01 for the occipital region of the ≥80 day-PNA group). The hPod value for the posterior–temporal region was significantly smaller (more anti-phase) than those of the anterior–temporal region (*p* < 0.001 in both PNA groups) and the occipital region (*p* < 0.05 for the 40–79-day PNA group and *p* < 0.01 in the ≥80-day PNA group).

Overall, in the youngest age group (1–39 days PNA), more anterior regions exhibited an elevated in-phase pattern of hPod values. In the later age groups, the frontal region exhibited the least change toward an anti-phase pattern, whereas the posterior–temporal region exhibited the most pronounced shift toward an anti-phase pattern.

### 3.8 Differences in hPod values among clinical groups

**Fig. 9.**
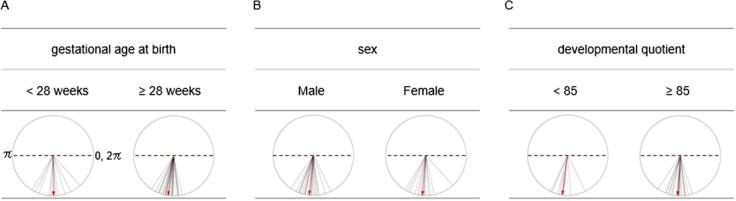
Comparison of hPod values between clinically categorized groups of preterm infants. Only recordings obtained after 40 days postnatal age were included, with a focus on recordings collected after the rapid changes in hPod values during early postnatal life. If multiple recordings from the same infant were available, only the youngest recording after the cutoff was used. Recordings were categorized based on gestational age at birth (A: <28 weeks [n = 12] and ≥28 weeks [n = 27]), sex (B: male [n = 23], female [n = 16]), and developmental quotient evaluated at 18 months corrected age (C: <85 [n = 9], ≥85 [n = 22]). The right edge of dotted lines shows in-phase (0, 2π) values, while the left shows anti-phase (π) values. Solid lines represent hPod values averaged across all channels in each recording during quiet sleep, and red arrows indicate group-averaged hPod values, calculated as the vector summation of the all-channel-averaged hPod values within each group. Group comparisons were conducted using the Watson–Williams tests. No significant differences were found among the groups.

Finally, hPod values were compared among clinical groups defined by GA at birth, sex, and DQ at corrected age 18 months. Given the rapid changes in hPod values within 40 days of PNA, only recordings obtained after 40 days of PNA were included in these analyses. No significant differences in all-channel-averaged hPod values were observed between the <28 weeks and ≥28 weeks GA groups, between female and male infants, or between those with DQ <85 and ≥85 (Fig. 9, *F*[1, 38] = 2.97, *p* = 0.19 for the GA groups, *F*[1, 38] = 0.039, *p* = 0.31 for the sex groups, and *F*[1, 30] = 0.47, *p* = 0.99 for the DQ groups). Subsequent analyses in each region also revealed no significant differences between these groups, except in the posterior–temporal region, where the <28 weeks GA group exhibited a significantly more in-phase pattern compared to the <28 weeks GA group (Supplementary Fig. S6).

## 4 Discussion

Our study offers several novel insights into the hPods of early infancy. First, we found that, within individual recordings, hPod values were comparable between AS and QS, as well as between homologous bilateral brain regions. Second, among preterm infants younger than term-equivalent age, the hPod values transition from an in-phase to an anti-phase pattern in a PNA-dependent manner. Finally, regional differences are noticeable during this developmental shift, with the temporal and occipital regions showing a pronounced transition to an anti-phase pattern as a function of PNA, while the frontal region tends to maintain an in-phase pattern.

The hPod values during AS and QS were highly consistent within each subject between 31 and 48 weeks PMA, but varied considerably across individuals. Our hypothesis, that hPod changes by sleep state, was not supported. We speculate that the resting-state phase difference between the oxy- and deoxy-Hb signals may reflect the operation of general physiological mechanisms related to oxygen transport and metabolism within the cerebral vascular system. Therefore, hPod values should be distinguished from the hemodynamic changes associated with neural activities and the functional brain networks evaluated in previous studies, which were believed to differ between AS and QS (Lee et al., 2020; Shiraki et al., 2024; Tokariev et al., 2019; Uchitel et al., 2023). Unlike sleep state-sensitive measures, hPod values offer a simpler index of brain development, calculated from recordings as short as 3 min regardless of behavioral state. This makes it suitable for use in clinically unstable or very preterm infants, including those requiring respiratory support. Thus, these values may serve as a practical biomarker for assessing vascular and metabolic brain development and may enable earlier, more accessible risk assessment in the NICU setting in the future.

Within our preterm cohort, including infants under 36 weeks of PMA, the hPod values exhibited a transition from in-phase to anti-phase patterns in a PNA-dependent manner rather than a PMA-dependent manner. This is supported by the fact that the hPod trajectory exhibited a stronger correlation with the PNA at the time of recording (Fig. 4). Also, the hPod values did not simply trend with PMA at the time of recording (Fig. 6). This is consistent with our previous findings that mainly assessed late preterm and term-born infants from 36 weeks PMA to approximately 6 months of age (Taga et al., 2018b; Watanabe et al., 2017). hPod values are influenced by hemodynamic and metabolic factors, such as the partial pressure of oxygen, partial blood volume, oxygen utilization rate, and the blood flow velocity, all of which change in conjunction with the development of the capillary network (Watanabe et al., 2017). A similar mechanism may underlie the changes observed in preterm infants younger than 36 weeks of PMA, particularly during the rapidly decreasing phase of hPod values, because circulatory maturation and the transition from fetal-to-adult Hb progress in a manner comparable to that in late preterm and term-born infants after entering the extrauterine environment (Bednarczuk et al., 2022). In addition, neural activity is essential for the development of vascular networks (Andreone et al., 2015), and this likely contributes to the gradual transition of hPod values after 2 months of PNA (Watanabe et al., 2017). Importantly, preterm infants experience the critical neurodevelopmental window of the third trimester in an extrauterine environment, which is often characterized by adverse stimuli (e.g., pain, noise, light) and diminished nurturing. Such altered conditions may trigger deviations in brain development compared to those of the typical intrauterine trajectories (Inder et al., 2023). Therefore, changes in hPod values after the period of rapid postnatal change may indirectly reflect abnormal neuronal activities during early life.

We also observed regional differences in hPod values. These became more anti-phase in the temporal and occipital regions compared to the frontal region, particularly after approximately 40 days of PNA, consistent with the well-established sequence of brain development. In general, the brain regions and networks responsible for primary sensory-motor functions (e.g., movement of extremities, auditory, and visual processing) tend to mature earlier than those involved in complex associative processing (Eyre et al., 2021; Gao et al., 2011). Additionally, brain myelination during early development proceeds in a posterior-to-anterior direction, beginning with the sensory-motor and visual tracts and culminating in the higher-order associative areas, including the poles of the frontal and temporal regions (Volpe & Inder, 2024). The anterior–temporal, posterior–temporal, and occipital channels of the present study are presumed to correspond anatomically to areas adjacent to the primary motor cortex, primary somatosensory cortex, and the superior part of the occipital lobe (Matsui et al., 2014; Shiraki et al., 2024), respectively, which are known to undergo earlier maturation than other regions. The trend is similar to that observed in full-term infants at 0, 3, and 6 months of age. The hPod values of the frontal region remain in more in-phase than those of the temporo-occipital regions (Taga et al., 2018b). These findings suggest that vascular and neuronal maturation proceed in parallel. Furthermore, as the posterior–temporal hPod values were more in-phase in preterm infants born <28 weeks of GA than in those of born ≥28 weeks of GA (Supplementary Fig. S6), changes in the external stimuli of the extrauterine environment may affect the development of vascular networks close to the primary somatosensory cortex.

When using continuous-wave fNIRS, as in the present study, it is difficult to determine accurately the absolute concentrations of oxy- and deoxy-Hb, or the extent of oxygen saturation, without considering the detailed optical properties of the head and individual and age-related variations. This has restricted the clinical applicability of fNIRS (Martini et al., 2024). In contrast, hPod, which represents the phase relationship between oxy-Hb and deoxy-Hb fluctuations, is less affected by differences in the absolute amplitude of signals (Watanabe et al., 2017). Furthermore, the present study showed that the hPod values were very consistent among homologous regions within the same individual, and remained stable across the various sleep states (Figs. 2 and 3, and Supplementary Fig. S4). In addition, the hPod values gradually shifted from in-phase to anti-phase as the PNA at the time of recording increased, not only in the cross-sectional group data but also within individual longitudinal data (Fig. 4). These characteristics suggest that hPod may serve as a sensitive indicator of individual structural and functional brain maturation, even when measured over a short duration in only a few brain regions and without distinguishing among sleep states.

Several limitations must be noted. First, although hPod values may indeed well-predict future neurodevelopmental outcomes, this study does not confirm such an association. This limitation is attributable to the characteristics of our preterm cohort, which did not fully mirror the diversity of preterm populations reported in previous international studies (Bell et al., 2022; Haga et al., 2023; Logan et al., 2025). Specifically, our participants tended to exhibit relatively stable clinical courses and favorable neurodevelopmental outcomes, because inclusion required the capacity to tolerate more than 30 min of EEG–fNIRS recording using an eight-channel NIRS device that covered the head. Obviously, infants requiring invasive respiratory support were excluded. To clarify the potential clinical utility of hPod values in terms of individual assessment, larger datasets including high-risk infants should be further investigated. Nevertheless, our high-precision recording setup enabled the evaluation of sleep-state and regional differences in hPod values. Second, data on late preterm and term-born infants were restricted at earlier PNAs compared to early preterm infants, as post-discharge recordings were logistically challenging in our setting. Although our NIRS channel placements and recording methods differed slightly from those in previous research (Watanabe et al., 2017), the developmental trajectory of hPod values in early preterm infants remained consistent. Therefore, we infer that, in late preterm and term-born infants, hPod values likely continue to decline over time, shifting toward a more anti-phase pattern with age, in contrast to early preterm infants. This difference may display the alteration of neurovascular coupling development in early preterm infants. Third, we did not record the types of blood vessels studied, which may part-explain the differences in hPod values. The arteries are thought to be the primary contributors to in-phase changes in oxy- and deoxy-Hb signals; capillaries and veins may contribute more to anti-phase changes (Boas et al., 2008; Fantini, 2002). Accordingly, hPod values may vary by the vascular composition of the measured tissue (Watanabe et al., 2017). Although it is not easy to obtain detailed information on the spatial distribution of blood vessels in each infant, a measurement region with an inter-optode distance of 2 cm is sufficient to include the cortical plate. Indeed, in young infants, it has been shown that a 2-cm inter-optode distance is adequate to detect hemodynamic responses associated with stimulus-evoked neural activity (Taga et al., 2007). Finally, we used group rather than multiple regression analysis to evaluate the hPod values, because these are angular data. Multiple regression systems for scalar values cannot be directly applied to angular data; conventional linear models are not appropriate. Specialized statistical approaches are needed. Accordingly, in this study, the recordings were grouped by parameters such as age, and circular statistical tests then used to examine whether the hPod values differed significantly across the groups. Although factors that might confound the links between hPod values and the PMA or PNA at the time of recording may exist, we found that only the age at the time of recording explained the differences in hPod values among the groups.

## 5 Conclusions

We observed a developmental shift in hPod values in preterm infants, transitioning from an in-phase to an anti-phase pattern between 31 and 48 weeks of PMA. The hPod values were comparable between AS and QS, and homologous bilateral brain regions within individuals. The temporal and occipital regions displayed a more pronounced anti-phase pattern than the frontal regions, indicating region-specific trajectories of brain maturation. Together, the findings suggest that the hPod value is sensitive to age- and region-related aspects of early neurovascular maturation. Thus, hPod may represent a unique and noninvasive bedside biomarker of early brain development, although further studies—particularly in more vulnerable, high-risk infants—are needed to establish the utility for predicting neurodevelopmental outcomes.

## Supplementary Material

Supplementary Material

## Data Availability

Data and the code used in this manuscript would be available from the corresponding author (H.K.) upon reasonable request.
