# Supplementary material for: Assessment of hPod measured by fNIRS as an indicator of brain development in preterm infants
Source: Imaging Neurosci (Camb). 2026 May 18;4:IMAG.a.1230. doi: 10.1162/IMAG.a.1230 (PMC13185623; doi:10.1162/IMAG.a.1230)
Supplement: Supplementary Material [file IMAG.a.1230_supp.pdf]

SUPPLEMENTARY MATERIALS for Assessment of hPod measured by fNIRS as an indicator of brain development in preterm infants

Anna Shiraki[1], Hama Watanabe[2], Takafumi Ushida[3], Ayano Yanagisawa[1], Misa Hashimoto[1], Misae Yamada[1], Hajime Narita[1], Takamasa Mitsumatsu[1], Ryosuke Suzui[1], Masahiro Kawaguchi[1], Yuji Ito[1], Hiroyuki Yamamoto[1], Tomohiko Nakata[1], Yoshiaki Sato[4], Jun Natsume[1,5], Gentaro Taga[2], Hiroyuki Kidokoro[1*]

Affiliations

[1] Department of Pediatrics, Nagoya University Graduate School of Medicine, Nagoya, Japan

[2] Graduate School of Education, The University of Tokyo, Tokyo, Japan

[3] Department of Obstetrics and Gynecology, Nagoya University Graduate School of Medicine, Nagoya, Japan

[4] Division of Neonatology, Center for Maternal-Neonatal Care, Nagoya University Hospital, Nagoya, Japan

[5] Department of Developmental Disability Medicine, Nagoya University Graduate School of Medicine, Nagoya, Japan

11 pages, 6 supplementary figures and 2 supplementary tables.

***Corresponding author**

Hiroyuki Kidokoro, M.D., Ph.D.

Address: 65 Tsurumai-cho, Showa-ku, Nagoya, Aichi 466-8550, Japan

Tel: +81-52-744-2294

Fax: +81-52-744-2974

E-mail: kidokoro.hiroyuki.i6@f.mail.nagoya-u.ac.jp

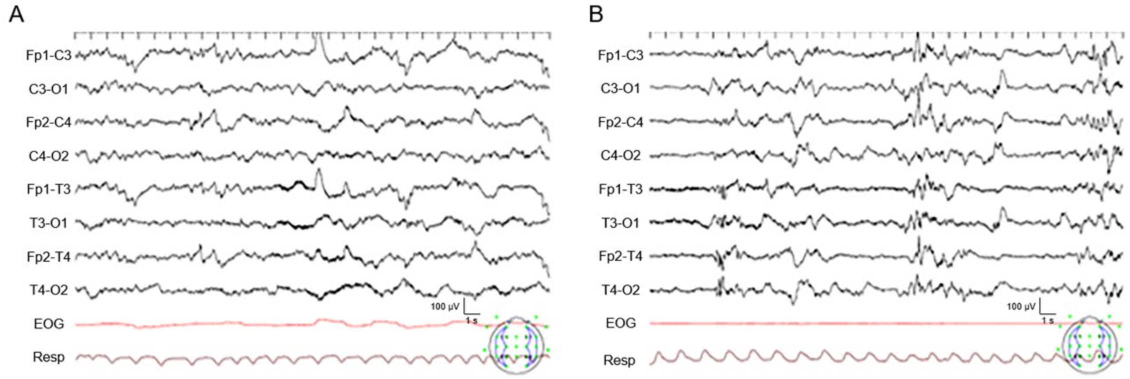

Supplementary Fig. S1.

Representative 30-second EEG polygraphy during (A) active sleep and (B) quiet sleep at 39.9 weeks of postmenstrual age. (A) Mixed patterns in terms of EEG, rapid eye movements in the electrooculogram (EOG), and irregular respiration (Resp). (B) *Tracé alternant* patterns in the EEG, no rapid eye movements in the EOG, and regular respiration. Other EEG patterns of AS include continuous or semi-continuous activity in the preterm period, and low-voltage irregular patterns at term-equivalent age. The QS patterns include *tracé discontinu* patterns in the preterm period and high-voltage slow patterns at term-equivalent age.

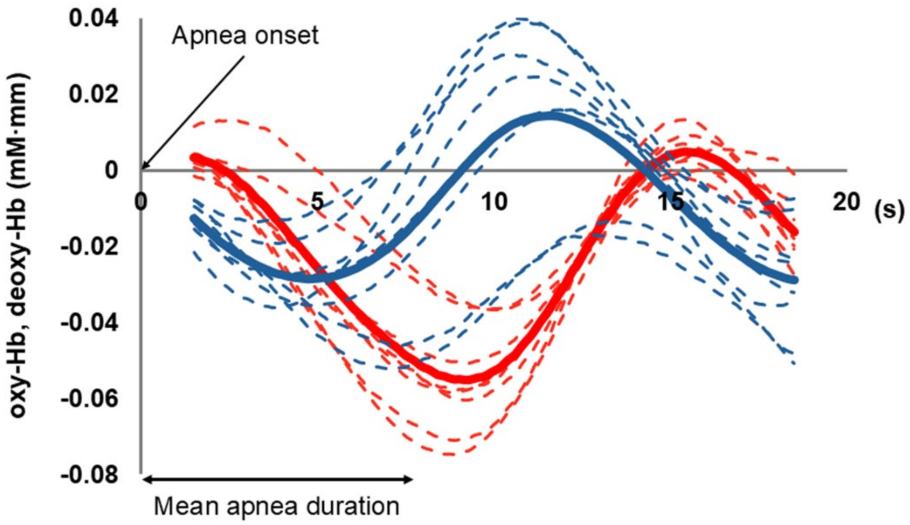

Supplementary Fig. S2.

Twenty-second grand averages of oxy- and deoxy-hemoglobin (Hb) signals of 22 periodic breathing apneas in one representative recording during quiet sleep. Red lines represent oxy-Hb and blue lines deoxy-Hb. Dashed lines indicate the 3-s averages in each channel, and solid lines the grand averages of the 3-s averages across all eight channels. The mean apnea duration was 7.7 s.

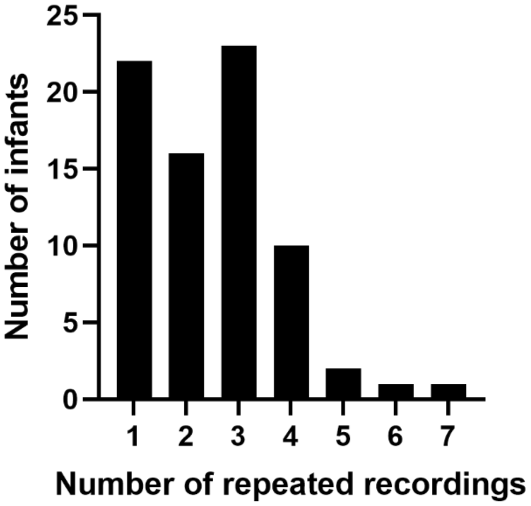

Supplementary Fig. S3.

The distribution of the number of repeated recordings per infant in the preterm group. In total, 186 recordings were obtained from 75 infants.

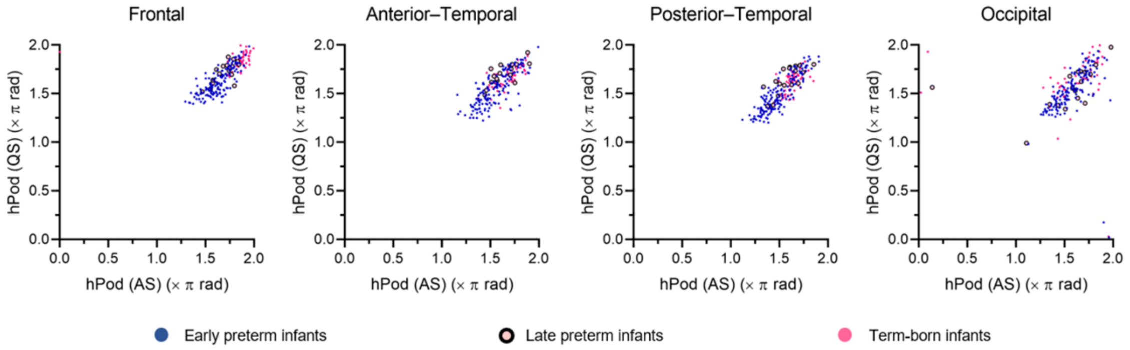

Supplementary Fig. S4.

Scatter plots of hPod values during active sleep (AS) and quiet sleep (QS) across different brain regions, based on a total of 221 recordings from early preterm, late preterm, and term-born infants. The hPod values were computed as averages across bilateral homologous channels in the frontal, anterior–temporal, posterior–temporal, and occipital regions. If data were available from only one side, that value was used for the analysis. Note that hPod values are circular, with 0π and 2π representing the same value. Correlations were assessed using the circular–circular correlation coefficient.

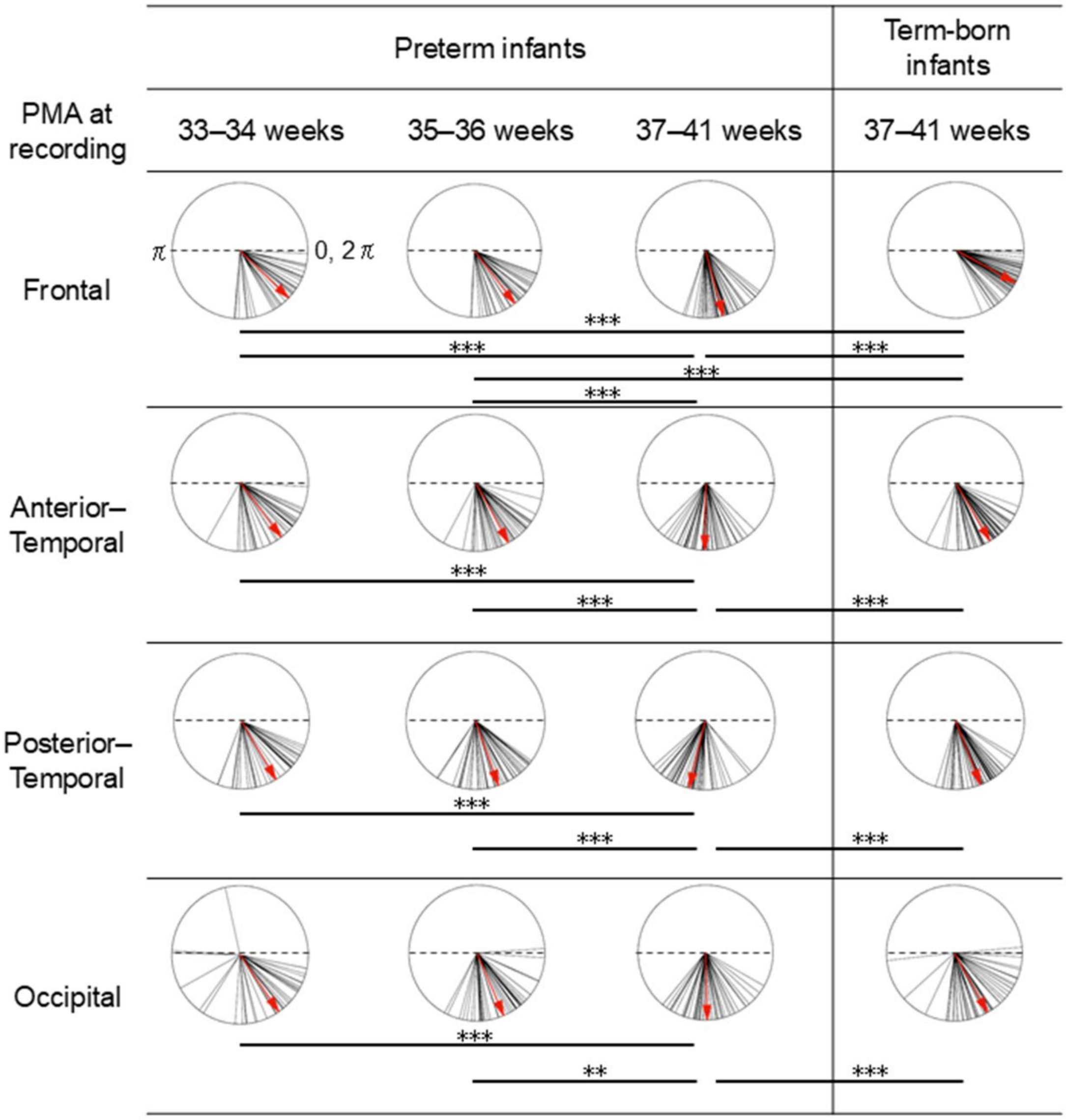

Supplementary Fig. S5.

Comparison of hPod values based on postmenstrual age (PMA) at the time of recording in each region. Group categorization is the same as in Fig. 5. The right edge of dotted lines shows in-phase (0, 2π) values, while the left shows anti-phase (π) values. Solid lines represent hPod values averaged across bilateral homologous channels in each recording during quiet sleep, and red arrows indicate group-averaged hPod values, computed as the vector summation of the bilaterally averaged hPod values within each group. $F[3, 179] = 51.37$, $p < 0.001$ for the frontal region; $F[3, 179] = 28.19$, $p < 0.001$ for the anterior-temporal region;

$F[3, 179] = 32.93$, $p < 0.001$ for the posterior-temporal region; and $F[3, 179] = 8.76$, $p < 0.001$ for the occipital region. Group comparisons were conducted using a Watson–Williams test, followed by the Tukey–Welsch *post-hoc* procedure. **$p < 0.01$, ***$p < 0.001$.

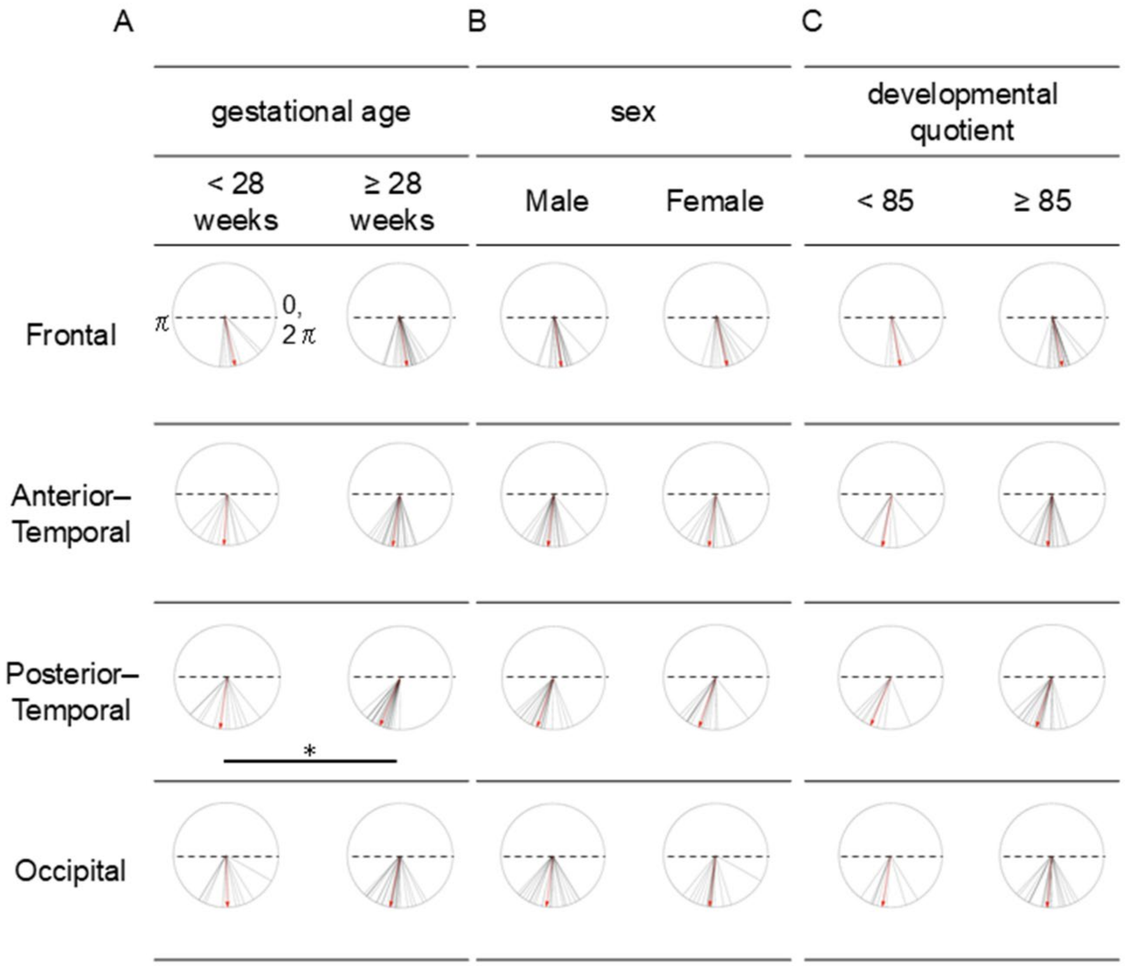

Supplementary Fig. S6.

Comparison of hPod values in different brain regions among clinically categorized groups of preterm infants. Only recordings obtained after 40 days of postnatal age were included, with a focus on recordings collected after the rapid changes in hPod values during early postnatal life. If multiple recordings from the same infant were available, only the youngest recording after the cutoff was used. Group categorization is the same as in Fig. 8. The right edge of dotted lines shows in-phase (0, 2π) values, while the left shows anti-phase (π) values. Solid lines represent hPod values averaged across bilateral homologous channels in each recording during quiet sleep in the frontal, anterior–temporal, posterior–temporal, and occipital regions. Red arrows indicate group-averaged hPod values,

computed as the vector summation of the bilaterally averaged hPod values within each group. $F[1, 38] = 6.12$, $p = 0.036$ for the posterior–temporal region between the gestational age groups (A). Group comparisons were conducted using the Watson–Williams test. *$p < 0.05$.

Supplementary Table S1. Characteristics of the postmenstrual age groups.

	Preterm infants			Term-born infants	
	33–34 weeks (n = 36)	35–36 weeks (n = 43)	37–41 weeks (n = 47)	37–41 weeks (n = 54)	*p*-value
GA at birth (weeks)	32.4 (24.6–34.0)	32.7 (24.6–34.9)	30.9 (24.6–34.9)	38.7 (37.0–40.7)	< 0.001
				***	
			***		
		***			
Female	14 (39 %)	18 (42 %)	21 (45 %)	30 (56 %)	0.39
PMA at the recording time (weeks)	34.1 (33.0–34.9)	35.9 (35.0–36.9)	38.1 (37.0–39.6)	39.2 (37.6–41.3)	< 0.001
	**	***		**	
		***			
			***		
		***			
PNA at the recording time (days)	12.5 (2–64)	21 (6–85)	49 (19–96)	2 (1–9)	< 0.001
		**		***	
		***			
			***		
		***			
Analytical duration during QS (min)	16.5 (3.0–33.0)	17.0 (3.5–49.5)	17.5 (3.5–38.5)	23.25 (10.5–48.0)	< 0.001
				*	
			***		
		**			

Values are shown as medians (ranges) or numbers (%).

GA, gestational age; PMA, postmenstrual age; PNA, postnatal age; and QS, quiet sleep.

The Kruskal-Wallis test was used to compare GA at birth, PMA at the recording time, PNA at the recording time, and the analytical duration during QS; and the chi-squared test was used to compare the sex ratio. *p < 0.05, **p < 0.01, ***p < 0.001 after Bonferroni correction for multiple comparisons.

Supplementary Table S2. Characteristics of the postnatal age groups in early preterm infants.

	1–39 days (n = 46)	40–79 days (n = 30)	≥ 80 days (n = 16)	*p*-value
GA at birth (weeks)	32.4 (27.3–33.9)	30.1 (24.6–33.7)	26.7 (24.6–32.9)	< 0.001
	**	*		
	***			
Female	21 (46 %)	15 (50 %)	3 (19 %)	0.10
PMA at the recording time (weeks)	34.1 (31.0–37.0)	38.1 (33.7–40.1)	39.2 (36.7–48.1)	< 0.001
	***			
	***			
PNA at the recording time (days)	13 (3–38)	51 (40–79)	87.5 (80–129)	< 0.001
	***	*		
	***			
Analytical duration during QS (min)	15.5 (3.0–33.0)	15.25 (3.5–32.5)	17.75 (10.0–31.5)	0.73

Values are shown as medians (ranges) or numbers (%).

GA, gestational age; PMA, postmenstrual age; PNA, postnatal age; and QS, quiet sleep.

The Kruskal-Wallis test was used to compare GA at birth, PMA at the recording time, and PNA at the recording time; one-way ANOVA was employed to compare analytical durations during QS; and the chi-squared test was used to compare the sex ratio. *$p < 0.05$, **$p < 0.01$, ***$p < 0.001$ after Bonferroni correction for multiple comparisons.
